# Cytogenetic and mutational analysis and outcome assessment of a cohort of 284 children with de novo acute myeloid leukemia reveal complex karyotype as an adverse risk factor for inferior survival

**DOI:** 10.1186/s13039-021-00547-0

**Published:** 2021-05-19

**Authors:** Xi Chen, Xingjuan Wang, Hu Dou, Zhenzhen Yang, Junqin Bi, Yi Huang, Ling Lu, Jie Yu, Liming Bao

**Affiliations:** 1 Center for Clinical Molecular Medicine, Ministry of Education Key Laboratory of Child Development and Disorders, Chongqing Key Laboratory of Pediatrics, Chongqing International Science and Technology Cooperation Center for Child Development and Disorders, Children’s Hospital of Chongqing Medical University, Chongqing, China; 2 Center for Reproductive Medicine, Baoji Maternal and Child Health Hospital, Shanxi, China; 3 Department of Clinical Laboratory, Key Laboratory of Pediatrics in Chongqing, Children’s Hospital of Chongqing Medical University, Chongqing, China; 4 Department of Clinical Laboratory, Nanchong Central Hospital, Nanchong, Sichuan China; 5 Department of Laboratory Medicine, The Second Affiliated Hospital of Kunming Medical University, Kunming, China; 6 Chongqing Key Laboratory of Child Infection and Immunity, Ministry of Education Key Laboratory of Child Development and Disorders, China International Science and Technology Cooperation Base of Child Development and Critical Disorders, Children’s Hospital of Chongqing Medical University, Chongqing, 400014 China; 7 Department of Rheumatology, Huashan Hospital, Fudan University, Shanghai, China; 8 Department of Hematology and Oncology, Children’s Hospital of Chongqing Medical University, No. 136 Zhongshang 2nd Road, Chongqing, 400014 China; 9 Department of Pathology, School of Medicine, University of Colorado Anschutz Medical Campus, 12705 E. Montview Boulevard, Suite 400, Aurora, CO 80045 USA

**Keywords:** Acute myeloid leukemia, Childhood, Cytogenetics, Mutation, Survival

## Abstract

**Background:**

Acute myeloid leukemia (AML) is rare in children. Although complex karyotype (CK) defined as ≥ 3 cytogenetic abnormalities is an adverse risk factor in adult AML, its prognostic impact on childhood AML remains to be determined.

**Results:**

We studied the prevalence, cytogenetic and mutational features, and outcome impact of CK in a cohort of 284 Chinese children with de novo AML. Thirty-four (12.0%) children met the criteria for CK-AML with atypical CK being more frequent than typical CK featured with -5/5q-, -7/7q-, and/or 17p aberration. Mutational prevalence was low and co-occurrence mutants were uncommon. Children with CK-AML showed shorter overall survival (OS) (5-year OS: 26.7 ± 10.6% vs. 37.5 ± 8.6%, *p* = 0.053) and event-free survival (EFS) (5-year EFS: 26.7 ± 10.6% vs. 38.8 ± 8.6%, *p* = 0.039) compared with those with intermediate-risk genetics. Typical CK tended to correlate with a decreased OS than atypical CK (5-year OS: 0 vs. 33 ± 12.7%.; *p* = 0.084), and CK with ≥ 5 cytogenetic aberrations was associated with an inferior survival compared with CK with ≤ 4 aberrations (5-year OS: 13.6 ± 11.7% vs. 50.0 ± 18.6%; *p* = 0.040; 5-year EFS: 13.6 ± 11.7% vs. 50.0 ± 18.6%; *p* = 0.048).

**Conclusion:**

Our results demonstrate CK as an adverse risk factor for reduced survival in childhood AML. Our findings shed light on the cytogenetic and mutational profile of childhood CK-AML and would inform refinement of risk stratification in childhood AML to improve outcomes.

**Supplementary Information:**

The online version contains supplementary material available at 10.1186/s13039-021-00547-0.

## Background

Acute myeloid leukemia (AML) is a group of clonal hematopoietic neoplasms that are characterized by aberrations in maturation, proliferation, and survival in the stem and progenitor cell compartments. Childhood AML is a relatively rare disease that accounts for 15%–20% of acute leukemias in children. Despite considerable progress have made in treating children with AML, about 30% of patients still experience relapse and do not survive beyond five years [1, 2]. Identification of additional genetic biomarkers predicting prognosis in childhood AML is needed to improve outcomes.

Cytogenetic and molecular mutational features play an important role in AML risk stratification [3, 4]. Complex karyotype (CK), commonly defined as three or more chromosomal aberrations in the absence of the WHO recurrent genetic aberrations of t(8;21)(q22;q22), inv(16)(p13.1q22) /t(16;16) (p13.1;q22); t(15;17)(q22;q12); t(9;11)(p22;q23), t(6;9)(p23;q34), inv(3)(q21q26)/t(3;3)(q21;q26), and t(9;22)(q34;q11.2) [5–8], is considered as an adverse risk factor in adult AML, but its prognostic impact on childhood AML remains to be determined [5, 9]. Differences in AML genetic profile between adults and children are well documented [10–12], and their impact on outcome may differ among age groups [6, 13]. There is also evidence to suggest geographic heterogeneity of AML cytogenetic and molecular features worldwide [14–17]. So far, few studies have been focused on the cytogenetic and molecular profile of childhood CK-AML, largely owing to the rarity of AML in children and none in a Chinese population [18–20].

Here, we report a study that investigated the prevalence, features, and clinical correlation of cytogenetic and mutational characteristics of CK-AML in a cohort of 284 children with AML. Our study showed CK was associated with decreased survival in childhood AML and its impact on outcome correlated with the number of chromosomal aberrations. These results would aid in informing risk stratification of childhood AML to guide risk-adapted therapy.

## Materials and methods

### Patients and samples

A total of 284 patients (≤ 18 years old) with de novo AML were enrolled in the study between 2007 and 2018 at Children’s Hospital of Chongqing Medical University in China. The diagnoses were based on histological, cytogenetics, and immunophenotyping analyses of bone marrow. The patients were treated with daunorubicin/cytarabine/etoposide (DAE)-based regimen following the protocols of the Pediatric Hematology Group of Chinese Medical Association [21]. The study was reviewed and approved by the Ethics Committee of Children’s Hospital of Chongqing Medical University in accordance with the Declaration of Helsinki.

### Cytogenetic analysis

G-banded karyotyping and fluorescence in situ hybridization (FISH) studies were performed according to the standard procedures [22]. A complete study required analysis of at least 15 metaphase cells. The FISH probes included *RUNX1/RUNX1T1*/t(8;21)(q22;q22), *CEBP/MYH11*/inv(16)(p13.1q22) /t(16;16) (p13.1;q22); *PML/RARA*/t(15;17)(q22;q12); *BCR/ABL1/*t(9;22)(q34;q11.2), and *KMT2A*(*MLL*)/11q23 rearrangement (Abbott Molecular, Abbott Park, Illinois). A complex karyotype was defined as three or more chromosomal aberrations in the absence of the WHO recurrent AML genetic aberrations of t(8;21)(q22;q22), inv(16)(p13.1q22) /t(16;16) (p13.1;q22); t(15;17)(q22;q12), t(6;9)(p23;q34.1), *KMT2A(MLL)/*11q23 rearrangement, and t(9;22)(q34;q11.2) [19]. An unbalanced aberration involving two or more chromosomes was counted as two abnormalities [23, 24]. Down syndrome AML was excluded from the study. CK patients with -5/5q-, -7/7q-, and/or 17p aberrations were assigned as typical CK while the others were deemed as atypical CK. Karyotype designation was in accordance with the International System for Human Cytogenomic Nomenclature 2016 [25].

Risk classification was following the modified US Children Oncology Group (COG) AML risk stratification scheme: low risk features included t(8;21)(q22;q22), inv(16)(p13.1q22) /t(16;16) (p13.1;q22); t(15;17)(q22;q12), mutated *NPM1,*and/or biallelic mutated *CEBPA*; high-risk factors were -7, -5/5q-, t(6;9)(p23;q34), t(9;22)(q34;q11.2), inv(3)(q21q26.2)/t(3;3)(q21;q26.2), *KMT2A*/11q23 rearrangement except t(9;11)(p21.3;q23.3), and *FLT3*-ITD; and intermediate-risk features were those without the high or low-risk features [20, 23, 26].

### Gene mutation analysis

Total RNA was extracted from bone marrow samples using the Tiangen RNAprep Pure Blood Kit (Tiangen Biotech, Beijing, China), and used as the template for cDNA synthesis with the Reverse Transcription System (Promega, Fitchburg, WI). DNA fragments covering the mutational hotspots were polymerase chain reaction (PCR) amplified from cDNA following the conditions previous described [27–32]. The PCR products were analyzed by Sanger sequencing, and the PCR products containing mutations were repeated at least once to confirm the presence of the identified mutations. Some mutant PCR products were subcloned into the pBackZero-T Vector (TaKaRa Biotechnology Co., Dalian, China) for further sequencing. PolyPhen and SIFT programs as well as COSMIC database (release v89, 15th May 2019) were employed to predict the pathogenicity of variants [33, 34]. Gene mutation hotspots analyzed in this study included *FLT3* (exons 14–15), *NPM1* (exon 12), *WT1* (exons 7 and 9), *NRAS* (exons 1–2), *KRAS* (exons 1–2), *IDH1* (exon 4), *IDH2* (exon 4), *KIT* (exons 8, 10, 11 and 17), *CEBPA* (exon 1), *CCDN1* (exon 5), *ASXL2* (exons 11–12), *DHX15* (exon 3), *GATA2* (exons 4–6), and *DNMT3A* (exon 2) [27–32].

### Statistical analysis

Patient characteristics were compared using chi-square (χ^2^), Fisher’s exact, or Mann–Whitney U test, as appropriate. Complete remission (CR) was defined as bone marrow with less than 5% blasts and evidence of regeneration of normal hematopoietic cells. Overall survival (OS) was calculated from the date of diagnosis to death or last contact. Event-free survival (EFS) was the time between diagnosis and occurrence of the first event (i.e., failure to achieve complete remission, relapse, secondary tumor, or death of any cause). OS and EFS were estimated using the Kaplan–Meier analysis, and the differences were compared using the log-rank test. A *p* value of ≤ 0.05 (two-sided) was considered statistical significance. The analyses were performed with SPSS software package v17.0 (SPSS, Inc., Chicago, Illinois).

## Results

### Childhood CK-AML cytogenetics

Of 284 patients in the cohort, 225 (79.2%) cases showed clonal cytogenetic aberrations. One hundred forty-three were classified as low-risk, 109 intermediate-risk, and 32 high-risk. Thirty-four (12.0%) patients met the criteria for CK (Additional file 1: Table 1). Among the patients with CK-AML, seven were less than 2 years old and 27 were at two years or older, resulting in CK incidences of 20.6% and 79.4% in children younger than two years and older than two years, respectively. Nine CK cases were typical CK and 25 atypical CK. The average number of aberrations in the typical and atypical CK subgroups were 6 [4–17] and 4 [3–13], respectively, and the difference was statistically significant (*p* = 0.025). Sixteen CK cases harbored three to four abnormalities and the others had five or more aberrations. Multiple cytogenetic clones defined as two or more clones were observed in 17 (50%) patients with CK-AML.

### Clinical and gene mutation characteristics of childhood CK-AML

**Fig. 1:**
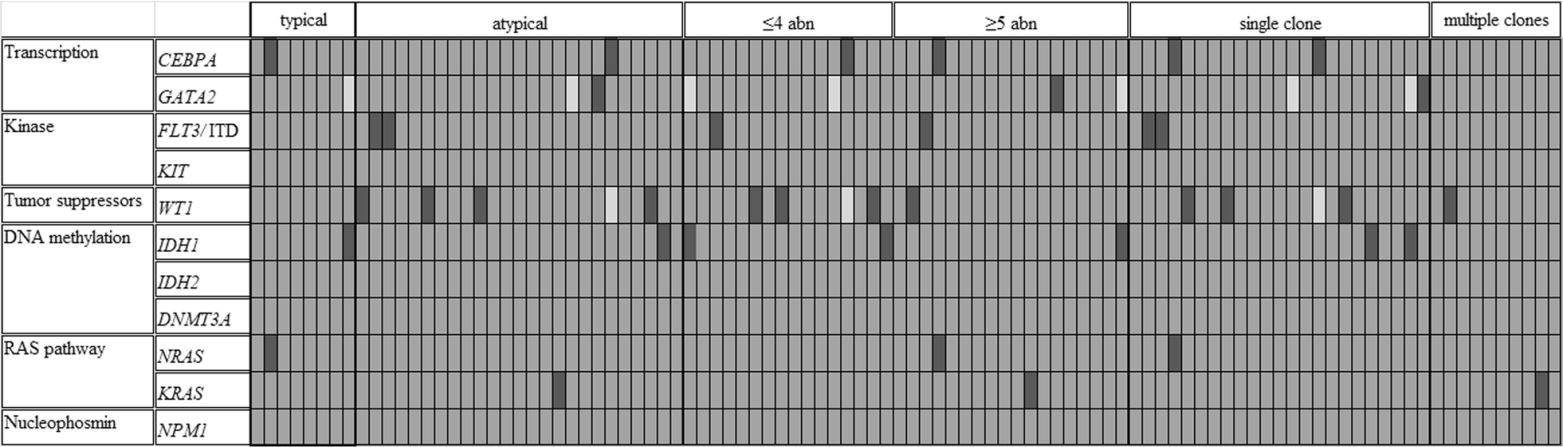
Distribution of gene mutations in CK-AML. Each column represents an individual case, and each row represents a single gene. Black denotes mutants; dark grey for wild-types; and light grey for mutation status not determined. CK: complex karyotype

There were no differences in clinical features between the CK and intermediate-risk groups except that patients with CK-AML tended to be younger (2.5 yrs. vs. 5.0 yrs., *p* = 0.031) (Table 1). Compared with children with CK-AML, patients with intermediate-risk features had a higher *NRAS* mutation incidence although the difference was not statistically significant (3% vs. 18%; *p* = 0.079) (Table 1). Among the patients with CK-AML, *WT1* gene had the highest mutational incidence (13%) followed by *CEBPA*, *FLT3/*ITD, and *IDH1* genes (6.0% each), and none was observed in *NPM1*, *KIT*, *CCND1*, *IDH2*, *ASXL2*, *DHX15*, and *DNMT3A* genes (Table 1). Patients with atypical CK-AML were likely to have higher blasts in bone marrow than those with typical CK-AML (73% vs. 68%; *p* = 0.08) while there were no differences in other clinical and molecular features between the two groups (Additional file 2: Table 2). Similar clinical and mutational features were observed between CK with ≤ 4 and ≥ 5 aberrations (Additional file 3: Table 3). CK-AML patients with a single clone were younger (2.0 vs. 3.0 yrs., *p* < 0.001) and had a higher percentage of blasts in marrow (70% vs. 68%; *p* < 0.001) compared to those with two or more clones, but no difference in gene mutational frequencies (Table 2). Of thirteen common AML genes examined in the CK-AML cohort, concomitant mutants were observed only in one patient (*CEBPA* and *NKRS*) (Fig. 1).

**Table 1:** Comparison of clinical and molecular features of childhood intermediate-risk AML and CK-AML

	CK (n = 34)	Intermediate-risk (n = 82)	*p*
Age (years)	2.5 (0.3–15.2)	5.0 (0.3–14.8)	0.031
Male/female	19/15	43/39	n.s
WBC (10^12^/L)	21.83 (0.88–249.96)	20.02 (0.85–389.96)	n.s
% blast in bone marrow	71 (20–95)	69 (16–97)	n.s
Gene mutations [% (no./total)]			
*CEBPA*	6 (2/33)	14 (10/74)	n.s
*FLT3/ITD*	6 (2/33)	0 (0/79)	n.s
* WT1*	13 (4/32)	13 (9/68)	n.s
* IDH1*	6 (2/33)	4 (3/74)	n.s
* KRAS*	3 (1/33)	5 (4/76)	n.s
* NRAS*	3 (1/33)	18 (14/80)	0.079
* KIT*	0 (0/33)	5 (4/76)	n.s
* IDH2*	0 (0/33)	4 (3/78)	n.s
* GATA2*	3 (1/31)	1 (1/69)	n.s
Others^†^	0	0	
Outcomes (%)			
Complete remission rate	60 (12/20)	83 (48/58)	0.076
Relapse	58 (7/12)	38 (18/48)	n.s
Relapse and failure to CR	60 (12/20)	48 (28/58)	n.s

^†^Genes include *ASXL2*, *DHX15*, *CCND1*, *NPM1*, and *DNMT3A.* CK denotes complex karypotype, and NK: normal karyotype, n.s.: no statistical difference

**Table 2:** Comparison of clinical and genetic features of childhood CK-AML with a single and multiple clones

	CK (single clone) (n = 17)	CK (≥ 2 clones) (n = 17)	*p*
Age (years)	2.0 (0.4–7.0)	3.0 (0.3–15.2)	< 0.001
Male/female	7/11	11/6	n.s
WBC (10^12^/L)	23.86 (0.88–152.86)	25.75 (1.33–249.96)	n.s
% blast in bone marrow	70 (20–93)	68 (31–95)	< 0.001
Gene mutations [%; (no./total)]			
* CEBPA*	7 (1/15)	6 (1/17)	n.s
* FLT3/ITD*	14 (2/15)	0 (0/17)	n.s
* WT1*	27 (4/15)	6 (1/17)	n.s
* IDH1*	0 (0/16)	12 (2/17)	n.s
* K-RAS*	0 (0/15)	6 (1/17)	n.s
* N-RAS*	0 (0/15)	6 (1/17)	n.s
* GATA2*	7 (1/15)	0 (0/16)	n.s
Other genes^†^	0	0	
Outcomes (%)			
Complete remission rate	67 (6/9)	67 (6/9)	n.s
Relapse	50 (3/6)	50 (3/6)	n.s
Relapse and failure to CR	67 (6/9)	89 (8/11)	n.s

^†^Genes include *MPN1*, *KIT*, *CCND1*, *CCND2*, *ASXL2*, *DHX15*, *DNMT3A*, and *IDH2*. CK denotes complex karypotype; n.s.: no statistical difference

### Impact of CK on childhood AML outcomes

**Fig. 2:**
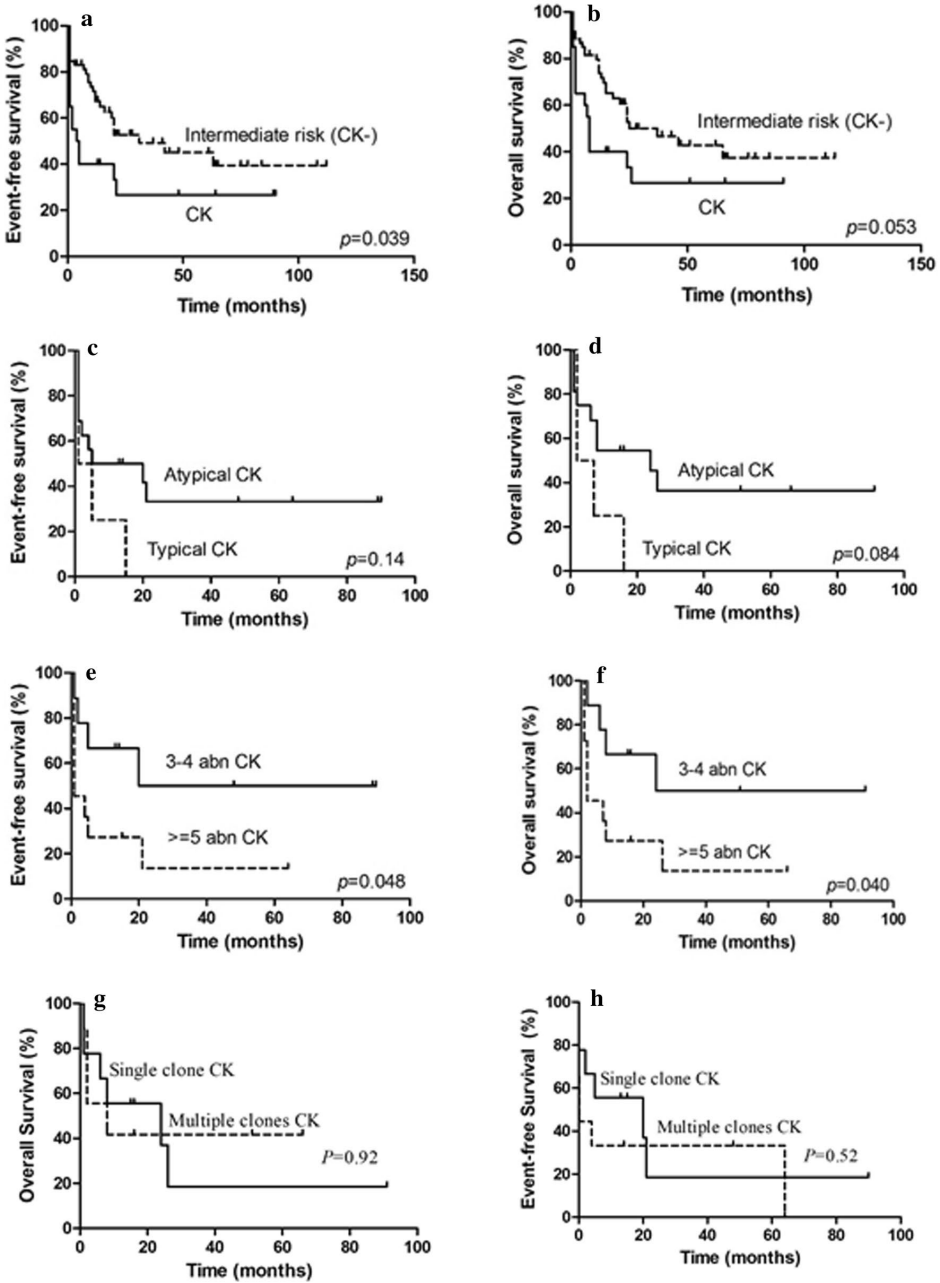
Comparison of overall survivals (OS) and even-free survivals (EFS) of AML with complex karyotype (CK) and intermediate-risk (**a** and **b**), typical and atypical CK (**c** and **d**), CK with ≤ 4 and ≥ 5 aberrations (**e** and **f**), and CK with a single and multiple clones (**g** and **h**). CK: complex karyotype; abn: abnormality

Outcome information was available from 60 patients in the intermediate-risk group and 20 patients in the CK group. The median follow-up was 15 months (range 1–91 months). Compared to those with intermediate-risk AML, patients with CK-AML seemed less likely to reach complete remission (60% vs. 83%; *p* = 0.076) (Table 1), and had a trend for shorter survivals (5-year OS: 26.7 ± 10.6% vs. 37.5 ± 8.6%, *p* = 0.053; 5-year EFS: 26.7 ± 10.6% vs. 38.8 ± 8.6%, *p* = 0.039) (Fig. 2). Children with typical CK-AML showed a trend for decreased OS (5-year OS: 0 vs. 33 ± 12.7%.; *p* = 0.084) but no difference in EFS (5-year EFS: 0 vs. 33.0 ± 12.7%; *p* = 0.14) compared with those with atypical CK-AML. Patients with ≥ 5 chromosome aberrations had an inferior OS and EFS (5-year OS: 13.6 ± 11.7% vs. 50.0 ± 18.6%; *p* = 0.040; 5-year EFS: 13.6 ± 11.7% vs. 50.0 ± 18.6%; *p* = 0.048) than ones with ≤ 4 aberrations, indicating a positive correlation between the number of chromosomal aberrations and worsen survivals. No differences in CR and relapse rates, as well as survivals, were observed between CK with a single and multiple clones (5-year OS: 18.9 ± 15.3% vs. 41.7 ± 17.3%; *p* = 0.92; 5-year EFS: 18.5 ± 16.1% vs. 33.3 ± 15.7%; *p* = 0.52) (Table 2 and Fig. 2). Outcome data were available on three CK-AML cases with mutant *WT1* gene, two relapsed, one died in 8 months and the other in 26 months. Another child was still alive at the last contact of six and half years after diagnosis.

## Discussion

In the past decades significant progresses have been made in treating childhood AML. But one-third of children with AML relapse and do not survive beyond 5 years [1, 2]. Cytogenetics is a major factor in AML risk classification which is important in guiding risk-adapted treatment [3, 4]. So far our knowledge of AML cytogenetics has been primarily derived from studies on adult AML and little is known about features and clinical correlation of CK in childhood AML, mainly owing to the rarity of the disease [18–20].

The prevalence of CK-AML in our study cohort, 12.0%, is comparable to 9.5% reported in another AML study in Chinese children [35]. In a study of children with AML in the United Kingdom, Harrison and colleagues observed a high CK incidence of 17.7%, but the study included CK with the WHO recurrent AML genetic aberrations such as t(8;21) and inv(16)(13). A relatively high CK prevalence, 18.5%, was also documented in a small study on Korean children [36]. In another study of 642 European children with AML, Rasche and colleagues reported a CK frequency of 9% [20]. In the present study, we also observed a distinct age-associated CK distribution with a higher incidence in toddlers than in young children and adolescents (20.0% in < 2 yrs. vs. 10.8% in ≥ 2 yrs). This is in agreement with the observation from a study of German children which showed similar distribution between the two age groups [37].

CK is considered an adverse risk factor in adult AML but its role in childhood AML remains inconclusive [3]. In our cohort most children with CK-AML reached CR, which is in line with the findings from other studies [20, 38, 39], but had shorter survivals compared with those with intermediate-risk features, demonstrating that CK is an adverse risk factor in childhood AML. Bager et al. reported reduced OS and EFS in children with CK-AML than those with non-CK-AML [18]. In their study, the comparator group, children with non-CK, also included ones with the t(8;21) and inv(16) which are associated with favorable outcomes. Therefore, it can’t definitively distinguish whether the improved survivals observed in non-CK group were due to the presence of the favorable cytogenetics in the comparator group or reduced survivals in the CK group were due to the worse effect of CK on AML than intermediate-risk cytogenetics in the comparator group. In another study of 59 children with CK-AML, Rasche and colleagues found no difference in survivals between children with CK-AML and those with either intermediate or low-risk features [20].

In adult CK-AML, shorter survival and a higher relapse rate have been associated with typical CK compared with atypical CK [19, 40]. We found no difference in EFS between typical CK and atypical CK groups among our patients but OS tended to be reduced in the typical CK-AML group. In a previous study of British children with CK-AML, Grimwade and colleagues described comparable outcomes between typical and atypical CK-AML [13]. In that study, the CK cohort also included the known favorable cytogenetics of t(8;21) and inv(16) which could influence the outcomes in either or both subgroups and thus, might be a confounding factor in assessing the impact of typical and atypical CK on AML outcomes [13]. Additional studies are necessary to further assess whether typical and atypical AML are two distinct disease entities with different outcomes in childhood AML. Furthermore, our study reveals CK with ≥ 5 aberrations is associated with shorter survivals than CK with ≤ 4 aberrations, suggesting a correlation of a higher number of chromosomal abnormalities with a worse prognosis. A similar relationship was also observed in childhood CK-AML in the study of Rasche and colleagues who reported significantly reduced OS among children with CK having > 5 aberrations compared to those with ≤ 5 abnormalities but no difference in EFS between the two groups [20]. More recently, Bager et al. observed a longer 5-year OS in CK with five or more aberrations and comparable EFS compared to CK with 3–4 aberrations [18]. Future studies are warranted to determine whether complex karyotypes with five or more chromosomal aberration is associated with worse outcome in childhood CK-AML.

In the present study, there were considerable number of CK cases harboring more than one cytogenetic clone but no differences in outcomes between ones with a single and multiple clones. Recent mutational studies using next-generation sequencing demonstrate that mutations at diagnosis play a critical role in leukemogenesis but mutational evolution during disease course is also important in influencing outcomes. These observations underscore the importance of continuous genetic profiling throughout the disease course in guiding optimal therapy to improve outcomes [41, 42].

Thus far, there is limited information on mutational profile of CK-AML and no mutational profiling of childhood CK-AML has been reported in the literature [19]. The results from our study show that atypical CK is more common than typical CK in childhood AML compared to high frequency of typical CK than atypical CK reported in adults [19, 40, 43]. Analysis of more than a dozen of common AML genes examined in our pediatric AML cohort showed that mutant incidences were low and concomitant mutants were rare. In a study of 81 genes in adult CK-AML, Mrozek and colleagues reported an average of two mutants per case [19]. Considering the fact that mutational frequencies in AML increase with aging, our results along with others demonstrate that molecular aberrations are uncommon in CK-AML [37]. Although the mutated *FLT3*/ITD and *IDH1* gene incidences in our childhood CK-AML cohort were comparable to those observed in adult counterparts, mutant *WT1* and *CEBPA* gene incidences were higher in our cohort than adult patients (*WT1*: 13.0% vs. 2.9%; *CEBPA*: 6.0% vs. 1.5%) [19]. Of three CK-AML patients carrying *WT1* gene mutation with outcome information available, two relapsed and died at 8 and 26 months, respectively, after diagnosis. These were similar to the observations reported by others that mutant *WT1* gene is associated with decreased survivals and high relapse [44, 45]. Finally, *TP53* gene aberrations have been reported in 40%-50% of adult patients with CK-AML [19, 46]. *TP53* mutational analysis was not performed in the present study, and we only observed one CK-AML case (2.9%) with a 17p13.1 deletion by cytogenetic analysis. Taken together, our results show a difference in cytogenetic and mutational profiles between childhood and adult CK-AML, which is in accordance with findings in other AML subtypes [37].

Differences in results between our study and others are likely attributed to variation in the composition of study cohorts including the number of patients, age, treatment modalities, criteria for complex karyotype (≥ 3 vs. ≥ 5 aberrations), geographic locations, ethnic groups, methods used in mutation analysis, and the number of genes examined. Our results need to be validated by future studies of large cohorts of children with CK-AML.

## Conclusions

To the best of our knowledge, no such studies have been reported in the literature and ours is the first in the Chinese population. Our results demonstrate for the first time that among Chinese children with CK-AML, atypical CK was more frequent than typical CK, mutational incidences were low and concomitant mutants were uncommon. CK-AML had reduced EFS and OS compared with intermediate-risk AML, indicating CK as an adverse risk marker for childhood AML. Typical CK-AML tended to correlate with decreased OS compared to atypical CK-AML. Moreover, CK-AML with five or more cytogenetic aberrations was associated with inferior survivals than CK with four or fewer abnormalities, suggesting that the number of cytogenetic abnormalities in CK may influence outcome. Results from our study would inform refinement of risk stratification for childhood AML to improve outcomes.

## Supplementary Information

**Additional file 1**. List of complex karyotypes

**Additional file 2**. Comparison of clinical and molecular features of childhood CK-AML with typical and atypical CK

**Additional file 3**. Comparison of clinical and molecular features of childhood CK-AML with ≤4 or ≥5 cytogenetic aberrations

## Data Availability

All relevant data and materials are included in this publication.

## Abbreviations

AML: Acute myeloid leukemia
OS: Overall survival
EFS: Event-free survival
CK: Complex karyotype
FISH: Fluorescence in situ hybridization
DAE: Daunorubicin/cytarabine/etoposide
abn: Abnormality
PCR: Polymerase chain reaction
