# Supplementary material for: Cytogenetic and mutational analysis and outcome assessment of a cohort of 284 children with de novo acute myeloid leukemia reveal complex karyotype as an adverse risk factor for inferior survival
Source: Mol Cytogenet. 2021 May 19;14:27. doi: 10.1186/s13039-021-00547-0 (PMC8136172; doi:10.1186/s13039-021-00547-0)
Supplement: Supplementary file 1 [file 13039_2021_547_MOESM1_ESM.docx]

Case ID
1#
2
