# Supplementary material for: Cytogenetic and mutational analysis and outcome assessment of a cohort of 284 children with de novo acute myeloid leukemia reveal complex karyotype as an adverse risk factor for inferior survival
Source: Mol Cytogenet. 2021 May 19;14:27. doi: 10.1186/s13039-021-00547-0 (PMC8136172; doi:10.1186/s13039-021-00547-0)
Supplement: Supplementary file 2 [file 13039_2021_547_MOESM2_ESM.docx]

Supplementary Table 2. Comparison of clinical and molecular features of childhood AML with typical and atypical CK

	Typical CK (n=9)	Atypical CK (n=25)	*p*
Age (years)	3.0 (1.0-15.2)	2.0 (0.3-12.0)	n.s.
Male/female	6/3	13/12	n.s.
WBC (10^{12}/L)	16.6 (3.58-56.69)	25.8 (0.88-249.96)	n.s.
% blast in bone marrow	68 (20-83)	73 (31-95)	0.082
Gene mutations [%; (no./total)]			
CEBPA	13 (1/8)	4 (1/25)	n.s.
FLT3/ITD	0 (0/8)	8 (2/25)	n.s.
WT1	13 (0/8)	13 (3/24)	n.s.
IDH1	13 (1/8)	4 (1/25)	n.s.
KRAS	0 (0/8)	4 (1/25)	n.s.
NRAS	13 (1/8)	0 (0/25)	n.s.
GATA2	0 (0/8)	4 (1/24)	n.s.
other genes†	0	0	
Outcomes (%)			
complete remission rate	50 (2/4)	63 (10/16)	n.s.
relapse	0 (0/2)	40 (4/10)	n.s.
relapse and failure to CR	50 (2/4)	63 (10/16)	n.s.

Note: †genes include *NPM1*, *KIT*, *CCND1*, *ASXL2*, *DHX15*, and *DNMT3A*.CK denotes complex karyotype, n.s.: no statistical difference.
