# Supplementary material for: Cytogenetic and mutational analysis and outcome assessment of a cohort of 284 children with de novo acute myeloid leukemia reveal complex karyotype as an adverse risk factor for inferior survival
Source: Mol Cytogenet. 2021 May 19;14:27. doi: 10.1186/s13039-021-00547-0 (PMC8136172; doi:10.1186/s13039-021-00547-0)
Supplement: Supplementary file 3 [file 13039_2021_547_MOESM3_ESM.docx]

Supplementary Table 3. Comparison of clinical and molecular features of childhood CK-AML with ≤4 and ≥5 cytogenetic aberrations

	CK (≤4 abn) (n=16)	CK (≥5 abn) (n=18)	*p*
Age (years)	2.8 (0.3-12.0)	2.92 (0.3-15.2)	n.s
Male/female	9/7	10/8	n.s.
WBC (10^{12}/L)	21.50 (0.88-152.86)	21.83 (3.34-249.96)	n.s.
% blast in bone marrow	76 (31-93)	67 (20-95)	n.s.
Gene mutations [%; (no./total)]			
CEBPA	7 (1/15)	6 (1/18)	n.s.
FLT3/ITD	7 (1/15)	6 (1/18)	n.s.
WT1	21 (3/14)	6 (1/18)	n.s.
IDH1	7 (1/15)	6 (1/18)	n.s.
K-RAS	0 (0/15)	6 (1/18)	n.s.
N-RAS	0 (0/15)	6 (1/18)	n.s.
GATA2	0 (0/15)	6 (1/16)	n.s.
other genes†	0	0	
Outcomes (%)			
complete remission rate	75 (6/8)	50 (6/12)	n.s.
relapse	33 (2/6)	33 (2/6)	n.s.
relapse and failure to CR	50 (4/8)	67 (8/12)	n.s.

Note: †genes include *MPN1*, *KIT*, *CCND1*, *CCND2*, *ASXL2*, *DHX15*, and *DNMT3A*, and *IDH*. CK denotes complex karypotype; n.s.: no statistical difference.
